# Juvenile Spondyloarthritis: What More Do We Know About HLA-B27, Enthesitis, and New Bone Formation?

**DOI:** 10.3389/fmed.2021.666772

**Published:** 2021-05-20

**Authors:** Shi Huan Tay, Joo Guan Yeo, Jing Yao Leong, Salvatore Albani, Thaschawee Arkachaisri

**Affiliations:** ^1^SingHealth Duke-National University of Singapore Academic Medical Centre, Translational Immunology Institute, Singapore, Singapore; ^2^Duke-National University of Singapore Medical School, Singapore, Singapore; ^3^Rheumatology and Immunology Service, Department of Pediatric Subspecialties, KK Women's and Children's Hospital, Singapore, Singapore

**Keywords:** juvenile arthritis, spondyloarthritis, enthesitis related arthritis, HLA-B27, osteogenesis, enthesitis

## Abstract

Juvenile spondyloarthritis (JSpA) refers to a diverse spectrum of immune-mediated inflammatory arthritides whose onset occurs in late childhood and adolescence. Like its adult counterpart, JSpA is typified by a strong association with human leukocyte antigen-B27 (HLA-B27) and potential axial involvement, while lacking rheumatoid factor (RF) and distinguishing autoantibodies. A characteristic manifestation of JSpA is enthesitis (inflammation of insertion sites of tendons, ligaments, joint capsules or fascia to bone), which is commonly accompanied by bone resorption and new bone formation at affected sites. In this Review, advances in the role of HLA-B27, enthesitis and its associated osteoproliferation in JSpA pathophysiology and treatment options will be discussed. A deeper appreciation of how these elements contribute to the JSpA disease mechanism will better inform diagnosis, prognosis and therapy, which in turn translates to an improved quality of life for patients.

## Introduction

Juvenile spondyloarthritis (JSpA) is a heterogeneous group of inflammatory arthritides whose onset occurs before the age of 16. A diverse spectrum of disorders, JSpA is characterized by varying degrees of peripheral and axial arthritis, enthesitis, and a strong association with human leukocyte antigen-B27 (HLA-B27). Thus, JSpA not only includes children meeting the criteria for juvenile idiopathic arthritis (JIA) categories of enthesitis related arthritis (ERA), juvenile psoriatic arthritis (JPsA), but also juvenile ankylosing spondylitis (JAS), reactive arthritis and inflammatory bowel disease (IBD)-associated arthritis. The underlying etiology of JSpA remains unknown, though robust familial aggregation and increased risk in HLA-B27-positive individuals allude to significant genetic susceptibility interacting with a plethora of environmental triggers.

JSpA is a common rheumatic disease reported in children worldwide, of which ERA is the most prevalent at about 10 to 40% whereas JPsA represents ~2 to 10% of all JIA patients (1–7). JAS, on the other hand, accounts for 1 to 7% of children in national pediatric rheumatic disease registries in Europe and North America (8–11). While the disease course of JSpA is highly variable, patients (especially ERA relative to other JIA subtypes) tend to show a worse prognosis with greater pain, poorer physical function, and persistent disease activity (5, 12). Much of the morbidity may be attributed to unrelenting inflammation and excessive new bone formation at inflamed sites that eventually lead to loss of joint mobility. Even though biologics like anti-tumor necrosis factor (TNF) drugs have attained commendable success in reducing inflammation, only around 20% of patients achieve remission off medication within 4 to 5 years and radiographic progression persists in a significant number of treated individuals (13, 14). Thus, disentangling the causative relationship between inflammation (in particular at the entheses) and new bone formation as well as delineating the role of HLA-B27 in both processes are two critical gaps in knowledge. Addressing these questions will aid in identifying reliable prognostic biomarkers and therapeutic targets to limit inflammation and structural disease progression.

To better appreciate pathological new bone formation in JSpA, it is prudent to first understand how skeletal development and turnover take place under physiological conditions (15). There are two main types of osteogenesis: intramembranous and endochondral. Both commence with a mesenchymal tissue precursor, but they differ in how bone is formed and how they contribute to skeletal development. Intramembranous ossification sees direct differentiation of mesenchyme into bone, and is responsible for the development of flat bones (e.g., skull, mandible, and lateral clavicles). In contrast, endochondral ossification relies on chondrogenic differentiation whereby mesenchymal progenitors first condense into an “anlagen” before further differentiating into hypertrophic chondrocytes (16). Angiogenesis subsequently occurs and cartilage is progressively replaced by bone matrix synthesized by osteoblasts. Endochondral ossification steers the formation of the rest of the skeleton (i.e., axial and appendicular). In response to biological and biomechanical stimuli, bone homeostasis is maintained throughout life primarily via careful balancing of local bone resorption by osteoclasts and new bone formation by osteoblasts (17). While endochondral bone formation is crucial for skeletal growth and development, it can also be reactivated in pathological conditions such as fracture repair and local inflammation leading to syndesmophyte and enthesophyte formation (18, 19).

The structural hallmark of JSpA, comparable to its adult counterpart, is an altered bone remodeling process that paradoxically manifests as excessive new bone formation at inflamed peri-articular sites whilst in a background of increased systemic bone resorption (20, 21). How inflammation is associated with new bone formation remains an enigma, especially since inflammation classically promotes RANKL-mediated osteoclastogenesis and osteoclast activation to stimulate bone loss (22). Thus, several fundamental questions have dominated over the past decades: (1) if inflammation and new bone formation in JSpA are closely linked or uncoupled, (2) why and how inflammation co-localizes with new bone formation at entheseal sites, and (3) the contribution of HLA-B27 to these processes. Thus, this Review seeks to critically evaluate recent literature in both JSpA and its closely related adult SpA to resolve the aforementioned issues and appraise their translatability to improving diagnosis, prognosis, and therapy for JSpA patients.

## Deciphering the Link Between Inflammation and New Bone Formation In JSPA

The relationship between inflammation and new bone formation in JSpA remains disputed. Two schools of thought, based on key findings from studies on adult SpA, dominate the field. One espouses a direct and sequential link between inflammation and new bone formation, while the other advocates an uncoupling of both processes. Even though inflammation can manifest separately or concurrently as enthesitis and synovitis, this Review will focus on evidence regarding enthesitis as it is pathognomonic of SpA.

## Link Between Inflammation and New Bone Formation: Existing Ideas and Evidence

The first view proposes that active inflammation may temporarily act as a brake on bone repair or remodeling. Inflammation induces inhibitors of new bone formation and simultaneously promote erosive cartilage and bone destruction. As the initial inflammation resolves or fluctuates, osteoproliferation follows (23). However, new bone formation in JSpA is hypothesized to be an excessive form of physiologic bone repair, thereby resulting in the formation of syndesmophytes and enthesophytes, as well as ankylosis.

This concept is supported by early magnetic resonance imaging (MRI) studies as entheseal sites with prior inflammation tend to have an increased frequency of bony appositions (23, 24). Building upon these topographical associations, contemporary studies focused on the effect of TNF on SpA pathogenesis. Evidence from animal models informed how TNF-induced inflammation may regulate new bone formation in the entheses through some candidates, notably the Wingless-related integration site (Wnt) pathway. TNF, acting through the Wnt pathway inhibitor Dickkopf-related protein 1 (DKK-1), may be important in repressing new bone formation driven by Wnt signaling as demonstrated in human TNF-overexpressing murine models (25, 26). Thus, there may be a window of opportunity to halt new bone formation with early and sustained anti-TNF treatment, which should prevent significant bone destruction and preclude the excessive bone repair response. Also, late anti-TNF treatment may even accelerate new bone formation. However, findings from TNF inhibitor (TNFi) studies have cast aspersions on this hypothesis. In the DBA/1 mouse model of ankylosing enthesitis, TNF blockade with the soluble TNF receptor etanercept did not inhibit the formation of new cartilage and bone at the enthesis (27). In TNFi placebo-controlled trials, ankylosis neither accelerated nor slowed down in the TNFi-treated cohorts despite significantly reduced clinical signs and symptoms (28–30). All in all, it is difficult to conclude from these studies as apart from their conflicting results, the studies have critically overlooked other inflammatory mediators and also inconsistently used animal models.

The second perspective suggests that inflammation and new bone formation are simultaneously induced by common triggers (e.g., biomechanical stimuli, infection) but are uncoupled. Yet, interactions between signaling pathways involved are likely to be present (31). This hypothesis hinges upon the concept of entheseal stress, which entails mechanical load and microdamage at the entheses, in promoting inflammation and activating stromal progenitor cells for tissue remodeling and new bone formation. Therefore, ankylosis might not necessarily be a repair process initiated by bone damage, but more of a response to direct entheseal damage that may be partially dependent on chronic or recurrent inflammation.

Histological evidence supports this concept—syndesmophyte formation preferentially localized to the posterolateral vertebral rim where there is significant mechanical stress, and inflammation-associated bone loss and enthesophyte formation occurred at anatomically distinct regions of the Achilles tendon enthesis of SpA patients (32, 33). Furthermore, collagen-antibody-induced arthritis (CAIA) mice that were subjected to tail suspension had limited enthesophyte formation (34). Patients with radiographically advanced ankylosis might still suffer from persistent inflammation that is responsive to TNFi, and there are disorders (e.g., diffuse idiopathic skeletal hyperostosis i.e., DISH) that appear to rely on non-inflammatory-driven pathways of new bone formation (18, 35). Genetic and environmental factors could modulate the chronicity and intensity of inflammation as well as the extent of new bone formation, since ankylosis and its rate of progression are highly variable traits that do not correlate well with the degree of inflammation in SpA patients (18).

## New Data on the Link Between Inflammation and New Bone Formation

Advances in the field of osteoimmunology have shed light on the role of inflammation and its associated cytokines in the bone pathology of JSpA. Additionally, there is also a greater recognition of how local mechanical stress may promote disease.

### Interleukin-17/23

The interleukin (IL)-17/23 axis has been increasingly implicated in disease pathogenesis since early observations of increased serum levels of IL-17 and IL-23 alongside a T helper 17 (Th17) predominance in the circulation of SpA patients relative to healthy controls (36). IL-23, a cytokine primarily secreted by macrophages and dendritic cells, induces the production of IL-17 by T cells. IL-17 is pro-inflammatory and can synergize with TNF to orchestrate synovitis and enthesitis (37). However, their effects on bone physiology are unclear. In particular, IL-17 exerts a Janus-faced influence on bone formation as seen from various *in vitro* studies (38–45). IL-17 can stimulate osteoclastogenesis and block osteogenesis, but it may also promote bone-forming phenotypes in osteoblasts and their mesenchymal precursors under certain situations. Findings from animal and human SpA studies seem to favor IL-17A as a driver for bone formation, given that IL-17A enhances bone formation in C57BL/6 mice by stimulating the proliferation and osteoblastic differentiation of mesenchymal progenitor cells and it appears to do so via the JAK2/STAT3 pathway at least in adult AS patients (46, 47).

Recent studies have described entheseal resident immune cell subsets that can respond to IL-23 and produce IL-17A, which hint at pathological roles on exposure to appropriate triggers. In the CAIA mouse model of arthritis, IL-23 promoted highly specific entheseal inflammation reminiscent of SpA by acting on a distinct CD3^+^CD4^−^CD8^−^ IL-23R^+^RORγt^+^ entheseal resident T cell subset (48). Upon IL-23 stimulation, these T cells produced IL-17 and IL-22 of which the latter is likely to be important in bone remodeling. Osteoproliferative changes were reduced *in vivo* with anti-IL-22 administration and could be reproduced with systemic IL-22 overexpression. In healthy human donors, subsets of IL-17A-producing group 3 innate lymphoid cells (ILC3) and γδ T cells reside in the spinal entheses (49). Similarly, in newly diagnosed SpA patients, innate-like T cells possessing a Th17-skewed phenotype (RORγt^+^T-bet^lo^PLZF^−^ invariant NKT and γδ-hi T cell subsets) were enriched within inflamed sites, albeit in the joints rather than the entheses (50).

The importance of the IL-17/23 axis is further highlighted via studies investigating the effects of blocking IL-17 and IL-23. IL-17A knockout mice models displayed impaired bone regeneration and fracture repair at the femur when compared to wild-type mice (46). IL-17A inhibition concurrently reduced synovial inflammation (peripheral more than axial) and bone formation in animal models and peripheral SpA patients (51, 52). Surprisingly, in AS clinical trials, IL-17A inhibition (secukinumab, ixekizumab) was more effective than IL-23 blockade (ustekinumab, risankizumab) on spinal disease progression (42, 53, 54).

In summary, currently available evidence pinpoints the IL-17/23 axis as an integral component in SpA pathogenesis. The effects of the IL-17/23 axis may vary at different anatomical locations (i.e., peripheral vs. axial) owing to differences in biomechanical stress, which culminate in divergent molecular mechanisms of inflammation and bone remodeling. The preferential alleviation of spinal inflammation and ankylosis with IL-17A blockade in AS patients convincingly suggests that IL-17, not IL-23, is the major cytokine directing disease pathogenesis at least in axial SpA and that it is likely to be generated in an IL-23-independent manner. Indeed, there is evidence of an IL-23-independent pro-inflammatory loop incorporating Th17 autocrine IL-17 secretion induced by local prostaglandin E2 (PGE2) production, albeit in an *in vitro* rheumatoid arthritis (RA) system (55). Nonetheless, IL-23 overexpression in an HLA-B27-negative mouse model was still sufficient to trigger peripheral ankylosing enthesitis and appeared to bypass the requirement for mechanical overload, which signified that IL-23-dependent mechanisms may still be relevant in JSpA (48). While approximately a third of JSpA patients develop axial symptoms within several years of disease onset, peripheral disease is strongly associated with disease onset before 16 years of age (56). Thus, IL-23 could be critical especially in JSpA disease initiation and further research should focus on resolving this quandary of IL-23 dependence to inform therapeutic strategies.

The entheseal non-Th17 sources of IL-17A may be useful as prognostic and therapeutic targets, but their reliance on IL-23 induction and downstream functional roles have yet to be fully clarified. Additionally, these immune cell subsets are also rare and limited in tissue distribution, so this calls into question their contribution to disease initiation and progression. For instance, ILC3s were found not to be a major source of IL-17A in the joints of adult peripheral SpA patients (47, 57). On top of considering IL-17 production by those cells, it is also worthwhile to explore alternative sources since it is possible that IL-17A may be secreted from distant sites (e.g., in the gut) to influence synovial cells that may, in turn, be abnormally sensitive to the cytokine.

Regarding IL-23, its cellular origins in JSpA also require further delineation. Going against the traditional view of entheses being largely devoid of myeloid cells, a recent study identified in healthy human entheses and adjacent bone a resident CD14^+^ population that produces most of the inducible IL-23 (58). Peripheral monocytes isolated from patients with enthesitis also displayed increased IL-23 secretion following stimulation (58). It would thus be beneficial to make out how resident vs. tissue-infiltrating myeloid cells modulate IL-23 generation in inflamed entheses, albeit the pronounced difficulty in classifying myeloid subsets in tissues. Epithelial cells are also capable of secreting IL-23, which may portend gut, skin, and even lung involvement in the disease mechanism (59). It also remains to be seen how the remarkable polymorphism of the IL-23 receptor (IL-23R) functionally impacts JSpA pathogenesis, since various IL-23R single nucleotide polymorphisms (SNPs) have been strongly associated with AS, psoriasis, and IBD (58, 60, 61).

### TNF Superfamily

The role of TNF as a key driver of entheseal inflammation and its influence on bone pathology are definite. However, the contradictory effects of TNFi on radiographic progression and the inability of experimental models to accurately recapitulate SpA features substantially restrict how much we know about the cellular and molecular mechanisms by which TNF supports JSpA pathogenesis.

The TNFi conundrum may be partly explained by the duration for which patients were followed up after receiving anti-TNF therapy. Despite earlier placebo-controlled trials reporting that anti-TNF treatment was more effective in hampering inflammation than spinal radiographic progression in AS patients, they only had a 2 year follow-up period as it is unethical to expose patients to ineffective treatment for a longer duration. Recent studies have circumvented this hurdle by comparing TNFi-treated patients to biologics-naïve controls, and they indicated that prolonged TNFi therapy (especially for more than 4 years) may potentially slow down the progressive structural change in AS (62, 63). As such, this suggests that sustained TNFi treatment may confer beneficial effects on limiting inflammation and hence ankylosis.

Nevertheless, these results do not demonstrate total abolition of structural changes, so the extent of coupling between TNF and new bone formation in SpA remains equivocal. Given that inflammation is mediated by a plethora of factors (including the IL-17/23 axis) and not solely by TNF, more work is needed to delve into the relative contributions and interactions among such factors. Current anti-TNF literature has also focused more on axial rather than peripheral SpA, so this raises doubts concerning their impact on the peripheral disease-dominant JSpA. Conventional radiography, whilst favored to measure radiographic progression as the primary outcome for structural disease in human studies, has limited sensitivity to change (62, 64). A potential remedy may be to rely on alternative modalities such as high-resolution quantitative computerized tomography (HR-qCT) and whole-body MRI. These techniques should complement plain radiography by concurrently assessing enthesitis as well as its associated bone marrow oedema, bone erosion, and enthesophyte formation in peripheral SpA and JSpA (65, 66). Yet, age-related anatomical variations (e.g., physiologic subchondral oedema vs. pathological bone marrow oedema in children) may confound interpretations of inflammatory and structural lesions in JSpA, particularly in the absence of pediatric-specific definitions (56). Even so, the increased resolution of the entheseal organ and adjacent bony ultrastructure will prove invaluable in supplementing mechanistic studies to address why inflammation and new bone formation co-localize in JSpA.

The TNF-overexpressing experimental models, though commonly employed, have largely failed to phenocopy SpA. The human TNF transgenic (hTNFtg) and the TNFΔARE mice lack typical SpA features like spondylitis and enthesitis but instead develop destructive polysynovitis reminiscent of RA (25, 26, 34). Moreover, the latter has minimal endochondral bone formation and ankylosis at affected sites despite focal degradation of bone and cartilage (34). Despite these limitations, hTNFtg mice have been used to demonstrate the reversal of a destructive bone phenotype into a remodeling one characterized by new bone formation in synovial joints after blocking DKK-1, while TNFΔARE mice exhibited the suppression of peripheral enthesitis with reduction of mechanical strain. As such, these conclusions only partially reveal the causative relationship between inflammation (at least TNF-driven) and pathological new bone formation in JSpA.

A series of recent studies using another preclinical mouse model of TNF overexpression have shed light on how the structure of TNF may contribute to SpA pathogenesis (52, 67). The TgA86 mice systemically overexpress a mutant murine TNF gene that is defective at the ADAM17 cleavage site, thereby causing a specific increase of transmembrane-bound TNF (tmTNF) but not soluble TNF (sTNF) (68). Both tmTNF and sTNF are biologically active, but they may have varying affinities for the TNF receptors I and II (TNFRI and TNFRII) (69). The TgA86 mice appear more SpA-like: they not only develop significant peripheral arthritis but also enthesitis, spondylitis, osteitis, and endochondral bone formation leading to eventual axial and peripheral ankylosis. The same studies also demonstrated relative overexpression of tmTNF over sTNF in the synovial environment of SpA vs. RA patients, thereby hinting at probable mechanistic relevance. Indeed, it was found that stromal tmTNF overexpression could drive inflammation, especially at peripheral sites (52). Moreover, tmTNF-driven inflammation and bone erosion seemed to precede new bone formation, which involved both endochondral and membranous ossification (67). However, this model lacks extra-articular manifestations of the SpA disease spectrum, which suggests that additional triggers may be required to induce IBD, uveitis, and/or psoriasis in the background of increased susceptibility owing to the tmTNF-sTNF imbalance. It also proposes that the inability of TNFi to induce full inflammatory remission and arrest radiographic progression in many patients is due to the subpar blockade of tmTNF in a poorly-vascularized environment like the enthesis. Although illuminating, it is not entirely clear how a single cytokine can foment distinct pathologies. To address this question, we will need to determine how TNFRI and TNFRII are differentially activated, how tmTNF is differentially expressed across cells, as well as how TNF and other pro-inflammatory molecules (e.g., IL-17) interact in the entheseal microenvironment.

### Other Modulators of Inflammation

The intensity of inflammation may help to direct the switch between bone catabolism and anabolism in SpA. Constitutive low TNF levels, but not short-term or high-intensity TNF stimulation, induced persistent expression of Wnt proteins and downstream bone formation through NF-κB and JNK/activator protein 1 (c-Jun) signaling pathways *in vitro* (42). When either the canonical Wnt/β-catenin or non-canonical Wnt/protein kinase Cδ (PKCδ) pathway is inhibited, new bone formation is significantly suppressed *in vitro* and in mouse models of SpA. Thus, the findings add nuance to the TNF brake hypothesis: inflammation does not have to be completely resolved before osteoproliferation can commence, and prolonged subclinical inflammation may be detrimental to bone physiology at entheseal sites. This may explain why structural disease, fueled by sustained low-intensity inflammation, continues to progress in many patients receiving anti-TNF therapy. As such, there is merit in exploring the feasibility of an early, aggressive and constitutive anti-inflammatory treatment for better outcomes in JSpA. Concurrent efforts should also further look into the molecular workings of this putative relationship since we do not fully understand how different Wnt family members and other pro-inflammatory cytokines contribute to this process, as well as how other osteogenic pathways (e.g., bone morphogenetic proteins; i.e., BMPs) come into play. There may also be interindividual differences in the threshold for inflammation-mediated structural progression, which could help to explain why some patients are refractory to TNFi treatment.

The role of the calcium-sensing receptor (CaSR) has recently received close scrutiny, as CaSR^+^ osteoblasts were reported to have accumulated in entheseal sites of AS patients and animal models (70). Systemic administration of a CaSR antagonist NPS-2143 attenuated osteogenic differentiation *in vitro* and pathological new bone formation *in vivo*. Moving upstream, inflammation directly induced CaSR upregulation in osteoblasts through the NF-κB and JAK/Stat3 signaling pathways. Collectively, it appears that inflammation may directly promote osteogenic differentiation of precursor cells, on top of inducing the secretion of osteogenic growth factors, in the pathological bone-forming microenvironment. Yet, the study did not examine if non-osteoblast CaSR expression contributes to ankylosis and how flux in extracellular Ca^2+^ levels at inflamed entheses may alter CaSR activity to influence bone remodeling.

### Biomechanical Stress

As the entheses are essential for transducing mechanical forces to bone and providing stability, they are sites of concentrated stress. Their distinct microanatomy consisting of fibrocartilaginous tissue is well-adapted for the high mechanical demands, as it grants both stiffness and elasticity (32, 71). While it is possible to induce enthesitis with repeated mechanical overloading like during sports, such pathology is usually limited to one enthesis and resolves spontaneously. It has been proposed that excessive mechanical stress causes extracellular matrix damage and a rapid loss of mechanotransduction in tenocytes. Subsequently, a positive feedback loop of cell death, immune cell recruitment and production of pro-inflammatory molecules gives rise to sterile inflammation. When the stressor is removed, inflammation resolves and repair is collaboratively driven by immune and stromal cells (72).

However, SpA patients often suffer from long-term inflammation at multiple entheses, even though they may not report of significant trauma or mechanical strain at affected sites. Thus, SpA patients may possess a lower threshold for developing enthesitis reminiscent of an overexuberant response to stress (73). Moreover, the normal healing process at the entheses might be compromised by perturbed local and systemic immunity, which leads to persistent inflammation and ectopic bone formation. Contributing factors are likely to include genetics (e.g., IL-23R polymorphisms, HLA-B27) and environmental triggers (e.g., microbial infection and accompanying immune dysregulation), but the exact molecular mechanisms have yet to be determined (72). A study using TNFΔARE mice showed that hind limb unloading suppressed Achilles tendon enthesitis supposedly in a mitogen-activated protein kinase (MAPK) signaling-dependent but T cell-independent fashion, but it remains to be seen if the same mechanism can be demonstrated in other preclinical models and human patients (34). The impact of age-related differences in skeletal anatomy and kinematics on biomechanical stress has also been underexplored in JSpA. For example, could walking with adult-like velocity despite immature lower limbs (74) and aberrant tendon stiffening amidst increasing body mass during childhood (75) apply increased forces on the entheses of susceptible children to favor disease development?

Recognizing the significance of microtrauma in promoting site-specific inflammation, Benjamin and McGonagle proposed the concepts of “synovio-entheseal complexes” (SECs) and “functional entheses” to rationalize how stress concentrations may lead to simultaneous enthesitis, adjacent osteitis and synovitis in SpA (18). The SEC hypothesis captures the functional interdependence between entheses and synovial membrane in entheseal organs. As biomechanical stress at the entheses is dissipated, the secretion of pro-inflammatory factors from focal bony attachment sites may trigger secondary osteitis and synovitis. A functional enthesis refers to a region proximal to the bony attachment sites where tendons or ligaments wrap around bone pulleys. This region is similar to true fibrocartilaginous entheses in anatomy, biomechanics and pathology, and are well-documented sites of pathology in SpA. Even though the theories may explain the diffuse nature of enthesitis and provide a unifying biomechanical basis for SpA pathology, they are exceedingly difficult to prove in animal models and human patients as the contribution of autoimmunity cannot be easily debarred.

## Contribution Of Hla-B27 to Inflammation ind New Bone Formation

Possession of HLA-B27 is strongly associated with the development of JSpA, though adult-onset disease has a higher prevalence of HLA-B27 positivity (56). However, the exact pathogenic role of HLA-B27 is unknown despite intense investigation. Additionally, no more than 5% of HLA-B27^+^ individuals develop SpA, which suggests the involvement of other genetic and environmental influences.

## HLA-B27 in Inflammation: Driver Or Modulator?

There are several theories explaining how HLA-B27 can stimulate and sustain inflammation in SpA, and the most favored ones will be critiqued in greater detail alongside data that has recently emerged. In a nutshell, HLA-B27 may present arthritogenic peptides to cytotoxic T lymphocytes (CTLs), but B27 can also assume abnormal forms at the cell surface.

### Presentation of Arthritogenic Peptides

Like other major histocompatibility complex (MHC) class I molecules, HLA-B27's natural function is to present endogenous peptides to T cells, especially CTLs. In this process, cytosolic proteins are first degraded into peptide fragments and loaded onto HLA-B27. Next, the peptide-MHC complexes are transported to the cell surface where they interact with their cognate T cell receptors (TCRs) to elicit antigen-dependent, cell-mediated immune responses (76, 77). Thus, it was hypothesized that HLA-B27 could present arthritogenic peptide(s) to autoreactive CTLs, which may subsequently induce inflammation at target tissues in SpA (78). This was thought to be an undesirable consequence of prior infections, whereby microbial peptides elicit a CD8^+^ T cell response cross-reactive with combinations of HLA-B27 and self-peptides.

Several lines of evidence from human studies support this theory. Firstly, patients with reactive arthritis exhibited HLA-B27-restricted CD8^+^ T cell responses specific for causative bacteria, which may be possible triggers for SpA (79, 80). This finding is buttressed by the oligoclonal T cell expansion in the inflamed joints of SpA patients, which implies an ongoing antigenic-driven process (81, 82). Moreover, HLA-B27 subtypes possess unique peptide specificities owing to variation at the peptide-binding groove, so this provides structural evidence for the existence of distinct autoantigens in SpA (83).

Current studies have employed high-dimensional technologies to interrogate the specificity of the B27-restricted T cell responses, and to define putative autoantigens responsible for these responses. Using next-generation sequencing-aided high-throughput T cell receptor (TCR) repertoire analysis (Rep-seq), a recent study demonstrated a positive association between disease activity and the oligoclonal expansion of not just CD8^+^ but also CD45RA^+^ effector memory CD4^+^ (T_EMRA_; CD45RA^+^CD45RO^−^CD62L^−^) T cells in the inflamed joints of HLA-B27^+^ AS patients (84). It also identified common complementarity determining region 3 (CDR3) motifs unique to the pathological CD4^+^ and CD8^+^ T cells. Therefore, both T cell compartments appear critical for SpA pathogenesis, and they may be stimulated by shared arthritogenic autoantigens. Another study, via data-independent acquisition (DIA) and multiple reaction monitoring (MRM) mass spectrometry, identified 26 candidate autoantigens that are presented in less abundance by the protective HLA-B^*^27:06 and HLA-B^*^27:09 relative to six AS-associated variants (85).

However, evidence from animal studies is at odds with this hypothesis. The HLA-B27^+^ transgenic arthritic/colitis rat model persistently developed SpA-like features even after the depletion of CD8^+^ T cells with either anti-CD8α monoclonal antibodies or CD8α knockout (86, 87). Many murine models are also capable of phenocopying SpA to varying degrees in the absence of human HLA-B27, notably through the overexpression of human TNF (25, 26, 34, 52, 67). IL-23 overexpression alone in an HLA-B27^−^ background was also sufficient to drive murine SpA-like disease by activating IL-17-producing entheseal resident non-αβ T cells (48). These animal data complicate the significance of the arthritogenic peptide model in initiating disease. Even so, it was shown in HLA-B27/hβ2m–transgenic rats that B27-restricted T cell responses may augment entheseal inflammation preferentially through the IL-17/23 axis as IL-17A inhibition significantly diminished inflammation and new bone formation (88).

All in all, it is difficult to conclusively demonstrate pathogenic T cell responses to arthritogenic peptides from existing literature. New approaches to distinguish the specific T cell subsets at fault and the autoantigens that stimulate them will likely assist in this search, especially in light of the latest advancements in our understanding of antigen presentation. For instance, the proteasome-generated spliced peptide pool unexpectedly contributes to a significant portion of the MHC-I immunopeptidome (i.e., epitopes presented by MHC-I molecules), which suggests that there is a far greater antigenic peptide diversity with overlapping sequences derived from either human or pathogen proteomes (89). Moreover, the strong association of AS with endoplasmic reticulum aminopeptidase 1 (ERAP1), whose expression is restricted to HLA-B27^+^ individuals (90), argues for aberrant MHC-I antigen processing in the disease mechanism since ERAP1's only known function is to trim peptides prior to MHC-I presentation (91). Thus, future prediction tools must account for these complexities to optimize the set of candidate autoantigens to be tested for immunogenicity.

### Cell Surface Free Heavy Chain Forms

HLA-B27 normally exists as a heterodimer consisting of a polymorphic α-chain (encoded by the MHC gene) that is non-covalently linked to a non-polymorphic β_2_-microglobulin chain. Peculiarly, HLA-B27 can also assemble as cell surface free heavy chain (FHC) forms, such as the β_2_m-free, Cys67-mediated disulfide-bonded homodimers (B27_2_), on immune cells of SpA patients (92, 93). FHC molecules can elicit TCR-independent immune responses by binding to innate immune receptors on natural killer (NK), CD3^+^ T and myeloid cells (93). These receptors include the killer immunoglobulin receptors KIR3DL1, KIR3DL2, and LILIRB2 in humans (93) and the rodent paired immunoglobulin receptors (PIR) (80). In particular, the KIR3DL2/B27 interaction is of exquisite affinity and exerts pro-inflammatory effects by enhancing NK cell survival and CD4^+^ T cell proliferation in HLA-B27^+^ SpA patients (94). It is also associated with a Th17 phenotype in SpA: the circulation and inflamed joints of HLA-B27^+^ SpA and ERA patients were enriched with KIR3DL2^+^ CD4^+^ T cells (95), of which a subset produced IL-17 upon stimulation with B27_2_-expressing antigen presenting cells (96).

Nevertheless, more direct evidence on the function of these FHC forms in JSpA pathogenesis is needed. Future work has to explore the conditions favoring FHC and B27_2_ formation, the relationship between homodimer expression and JSpA disease severity, as well as the extent of protection afforded by inhibiting the KIR/B27 interactions.

### Other Theories

HLA-B27 can misfold in the endoplasmic reticulum (ER), which may lead to ER stress and the inflammatory unfolded protein response (UPR) (97). A possible consequence of the UPR is thought to be increased IL-23 production, which correlated with the degree of HLA-B27 misfolding in stimulated HLA-B27 transgenic rat bone marrow–derived macrophages (98, 99) and dendritic cells derived from healthy human volunteers (100). Yet, the failure of excess β2m in relieving ER arthritis in HLA-B27 transgenic rats (91) and the lack of direct evidence in human SpA (101) warrant caution.

HLA-B27 may also promote microbial dysbiosis to result in entheseal inflammation. There is evidence of local gut inflammation in more than half of AS patients and shared genetic associations between AS and IBD (102). The cecal microbiome is also altered in HLA-B27 transgenic rats (30). However, the jury is still out on how HLA-B27 alters the gut microbiome and promotes inflammation at distant sites.

## HLA-B27 in New Bone Formation: A Predisposing Factor?

HLA-B27 positivity has been associated with worse radiographic damage, more typical marginal syndesmophytes, and more frequent syndesmophyte symmetry in SpA patients (103). How does HLA-B27 specifically direct osteoproliferation in JSpA? The first line of evidence comes from the observation that HLA-B27^+^ individuals appear predisposed to exaggerated bone formation regardless of SpA disease status (104). This inflammation-agnostic osteoproliferative state may be attributed to increased Wnt signaling evident by lower serum concentrations of the Wnt pathway inhibitors DKK-1 and sclerostin (SOST), as well as elevated circulating levels of the ossification-enhancing Indian hedgehog (IHH). Yet, the molecular mechanisms by which these bone regulators exert and coordinate their effects in disease are not fully known.

A recent study suggests another possibility: HLA-B27 directly activates the tissue non-specific alkaline phosphatase (TNAP) pathway in mesenchymal stem cells (MSCs) to enhance bone mineralization and ectopic bone formation at inflamed entheses (105). RNA interference (RNAi)-mediated HLA-B knockdown reduced the expression of TNAP and its upstream transcription factor retinoic acid receptor-β (RARB), as well as the amount of mineralization, in MSCs derived from HLA-B27^+^ AS patients. Reciprocally, HLA-B27 overexpression by lentiviral transformation of control MSCs reversed the phenotype. Furthermore, TNAP blockade inhibited new bony appositions induced by AS MSCs that were implanted into surgically decorticated spinal sites of NOD-SCID mice. Collectively, HLA-B27 may encourage ectopic new bone formation in a TNAP-dependent manner. The study also suggests HLA-B27 misfolding as a potential trigger for this mechanism since HLA-B27 FHC forms substantially accumulated and the inositol-requiring 1 (IRE1)/spliced X-box–binding protein 1 (sXBP1) pathway was upregulated in AS MSCs. As such, the findings put forth a compelling paradigm directly implicating HLA-B27 in both inflammation and new bone formation. However, the relative importance of this mechanism in JSpA and how the entheseal stroma interacts with other players (e.g., immune cells, cytokines, and mechanical stress) to induce the HLA-B27-mediated TNAP pathway remain unclear.

Contrary to the previously discussed literature, a study argues against a direct role of HLA-B27 in mediating new bone formation in axial SpA (106). HLA-B27 overexpression in various *in vitro* mouse and human differentiation systems mimicking endochondral ossification did not result in differences in bone formation when compared to the HLA-B7 overexpression controls. While the experimental set-up was deliberately reductionist to exclude the influence of possibly intervening variables (e.g., pro-inflammatory cytokines, immune cells), its oversimplicity may render the study's conclusions premature.

In summary, there is sufficient evidence to surmise an ancillary role for HLA-B27 in pathological new bone formation, but how HLA-B27 specifically influences or signposts osteoproliferation deserves continued investigation. Future work should also aim to reconcile the pro-inflammatory and osteogenic capacities of HLA-B27.

## Integrating New to Old Data: Translating Findings to Clinical Practice for JSPA

**Figure 1:**
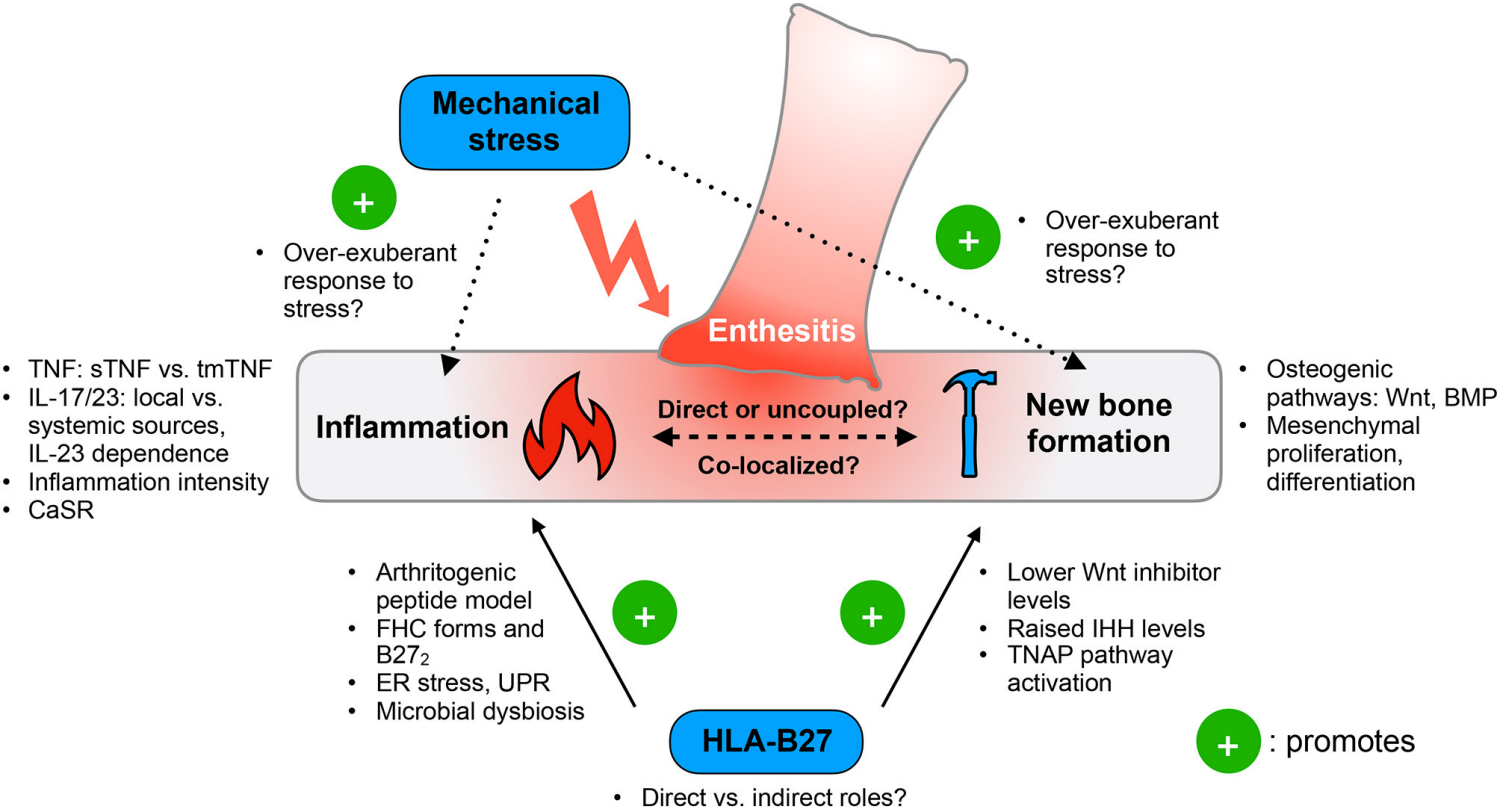
Functional model of ankylosing enthesitis in JSpA. Enthesitis is primarily driven by the tumor necrosis factor (TNF) and IL-17/23 pathways. Inflammation appears to influence osteoproliferation by acting on the Wnt and BMP pathways, as well as controlling mesenchymal proliferation and differentiation. The intensity of inflammation may determine the switch between bone loss and formation. However, it is still unclear if inflammation and pathologic new bone formation are directly linked or uncoupled. Mechanical stress has emerged as an important trigger of disease, yet the exact molecular mechanisms are unknown. HLA-B27 may contribute to both inflammation and pathologic new bone formation, but how crucial this genetic risk factor is to JSpA pathogenesis remains to be clarified. sTNF, soluble TNF; tmTNF, transmembrane TNF; CaSR, calcium sensing receptor; FHC, free heavy chain; B272, HLA-B27 homodimers; ER, endoplasmic reticulum; UPR, unfolded protein responses; Wnt, Wingless-related integrated site; IHH, Indian hedgehog; TNAP, tissue non-specific aminopeptidase; BMP, bone morphogenetic protein.

Taking a leaf from adult SpA, entheseal-specific bone turnover in JSpA is likely to depend on concomitant inflammatory and mechanical effects on bone physiology. Inflammation, by acting both directly and indirectly, drives bone loss and influences osteoproliferation. Genetic and environmental risk factors (e.g., HLA-B27, local biomechanical stress) may amplify the effects of inflammation at entheseal sites to promote new bone formation (Figure 1).

In JSpA, the treatment of enthesitis aims to resolve inflammation and prevent downstream inflammation-induced tissue responses. Non-steroidal anti-inflammatory drugs (NSAIDs) are the initial pharmacological management and can adequately control early disease as seen in adult AS patients (107), though no trials of NSAIDs have been reported in JSpA. NSAIDs may also hamper new bone formation since they inhibit PGE2, which possesses potent osteoinductive properties (108–110). When enthesitis becomes chronic and increases in severity, therapy is escalated to include disease-modifying anti-rheumatic drugs (DMARDs) and biological agents (111). The conventional DMARDs sulfasalazine and methotrexate have been shown to be effective for managing peripheral enthesitis in ERA and JAS patients (112, 113), but the same benefits may not extend to axial disease. As such, TNFi treatment is indicated for peripheral disease refractory to DMARDs or when axial disease is present since several trials in ERA have guaranteed its efficacy and safety (114).

Taking into account how inflammation pervades disease induction and progression, early and sustained anti-inflammatory treatment should form the bedrock of JSpA management. A sizeable number of patients have been unable to sustain disease remission despite receiving prolonged TNFi therapy (115–118). However, this refractoriness may be partly ascribed to (1) interindividual differences in the intensity threshold for structural progression, as well as (2) the contributions of other pro-inflammatory cytokines and local pathways. Thus, future treatment strategies demand greater personalization and combinatorial use of multiple therapeutic options, both pharmacological and non-pharmacological.

Promising therapeutic targets to complement TNF blockade include the IL-17/23 axis, especially in light of its purported role in both inflammation and new bone formation. Moreover, clinical trials of IL-17 and IL-23 inhibitors have been successful in reducing disease activity and structural progression in adult SpA. The therapeutic benefits of IL-17/23 inhibition are currently under evaluation in children, but it is expected that a similarly striking responsiveness will be replicated in JSpA. Whether or not synergy exists between anti-TNF and anti-IL-17/23 drugs on curbing JSpA is also a tantalizing prospect to follow up on. In addition, the recent discoveries of IL-17-producing entheseal resident innate-like T cells and ILC3s, as well as stromal tmTNF expression, draw attention to local mediators of inflammation in perpetuating disease. Coupled with the poor vascularity of the enthesis, it may be advantageous to assess how local anti-TNF and anti-IL17/23 treatment can dovetail systemic drug administration. An extension of the IL-17/23 axis is the Janus kinase (JAK)/STAT pathway, which is thought to activate the IL-17/23 axis so its blockade could augment the effects of IL-17/23 inhibition (119). An early trial using the JAK1/3 inhibitor tofacitinib has been moderately successful in reducing disease activity and radiographic disease in AS (120), but evidence is still sorely lacking in the JSpA realm.

The heightened awareness of local players in entheseal inflammation and new bone formation has also emphasized the advantages of relieving biomechanical stressors and targeting stromal remodeling pathways in JSpA. As JSpA patients may be predisposed to a pathologically exaggerated inflammatory response to mechanical stress, physical therapy could help by maintaining a safe loading environment for the entheses. Short-term interventions should aim to immediately reduce mechanical forces imparted to affected sites, such as through the use of custom-made foot and ankle orthotics (121, 122). On the other hand, long-term strategies ought to enhance the entheseal loading environment. Emulating the restorative treatment approaches of osteoarthritis (123), physical therapy in JSpA should concomitantly strengthen muscles of the limbs, trunk and back, as well as engage in neuromuscular training. Doing so will not only slow down the loss of range of motion (ROM) and correct poor functional positioning (114), but also minimize a major environmental risk for enthesitis to complement the action of anti-inflammatory drugs. Studies have also implicated various bone remodeling pathways (e.g., Wnt, BMP) in JSpA new bone formation, but pharmacological modulation of these processes (especially with systemic administration) may upset normal skeletal development (124, 125). As such, future studies are needed to assess feasibility and fine-tune therapeutic doses for maximal effectiveness.

HLA-B27 continues to be an important genetic risk factor for JSpA, but the molecular mechanisms behind this disease association remain at large. Nevertheless, progress has been made in clarifying the arthritogenic peptide model using high-dimensional technologies, and in identifying candidate pathways through which HLA-B27 exerts its influence on bone formation.

## Conclusion

JSpA is a largely inherited disease that is almost certainly influenced by ubiquitous environmental triggers. The recurrence of enthesitis, despite substantial heterogeneity in clinical presentation, emphasizes a common pathological process in a unique anatomical and immune microenvironment. This process is likely to depend on multiple steps consisting of initiation and augmentation of inflammation followed by local tissue responses leading to new bone formation. Current data provide strong support for the central roles of TNF, IL-17/23 axis, and HLA-B27 in JSpA pathogenesis, albeit incomplete evidence. Nevertheless, questions on temporality and gene-environment interaction in the relationship between inflammation and new bone formation remain, especially when the field is beset by inconsistencies in clinical trial design and deficiencies in preclinical disease models. Moving forward, one would have to integrate information from wide-ranging studies and clarify disease nomenclature. Doing so would be critical not just for translating findings in adult SpA to JSpA, but also for facilitating the otherwise tricky transition between pediatric and adult care.

## Author Contributions

ST, JY, and TA contributed to the conceptualization and writing of the article. JL and SA helped in the revision of the manuscript. All authors contributed to the article and approved the submitted version.

## Conflict of Interest

The authors declare that the research was conducted in the absence of any commercial or financial relationships that could be construed as a potential conflict of interest.
